# From data to innovation: how digital leadership cultivates employees’ data psychological ownership through perceived data value

**DOI:** 10.3389/fpsyg.2026.1922622

**Published:** 2026-08-28

**Authors:** Wei Yang, Yuru Hu

**Affiliations:** 1 School of Accounting and Finance, Anhui Finance & Trade Vocational College, Hefei, China; 2 School of Accounting, Anhui Wenda University of Information Engineering, Hefei, China

**Keywords:** conservation of resources theory, data asset psychological ownership, digital leadership, employee innovative behavior, perceived data value

## Abstract

**Purpose:**

Against the backdrop of the rapid development of the digital economy, stimulating employees’ data-driven innovative behavior has become a key issue in organizational management. This study aims to explore how perceived data value affects employee innovative behavior, the mediating role of data asset psychological ownership, and the moderating role of digital leadership.

**Methodology:**

Drawing on psychological ownership theory and conservation of resources theory, this study constructs a moderated mediation model with digital leadership as a contextual variable. Questionnaire data were collected from 279 full-time enterprise employees, and empirical tests were conducted using the PROCESS macro (Model 7).

**Findings:**

First, perceived data value is positively associated with employee innovative behavior. Second, data asset psychological ownership partially and significantly mediates the relationship between perceived data value and employee innovative behavior. Third, digital leadership positively moderates the link between perceived data value and data asset psychological ownership. Fourth, digital leadership also positively moderates the mediating effect of data asset psychological ownership; specifically, the indirect path from perceived data value to employee innovative behavior via data asset psychological ownership is reinforced when digital leadership is stronger.

**Originality:**

This study broadens the object scope of psychological ownership theory by extending it to data assets, and clarifies the psychological mechanism through which perceived data value shapes employee innovative behavior in digital settings. It also provides new micro-level psychological evidence for digital leadership research. Practically, the findings offer valuable implications for enterprises seeking to unlock employees’ innovative potential amid digital transformation.

## 1 Introduction

In the era of the digital economy, data has emerged as a vital production factor following land, labor, capital and technology. It serves as a core strategic resource for organizations to build sustainable competitive advantages (Akter et al., 2016; Wamba et al., 2017). International Data Corporation (IDC) projected that the global volume of data would exceed 175 zettabytes by 2025. Organizations across all industries are investing heavily in data infrastructure and digital transformation on an unprecedented scale. These efforts aim to fully unlock the strategic potential of data assets (Verhoef et al., 2021). The efficient utilization of data factors has become a core issue at the national strategic level. As primary agents who translate data into tangible value, employees and their data-driven behaviors are critical determinants of organizational innovation and sustainable development (Benitez et al., 2022; Fatima and Masood, 2024). Although the strategic value of data is widely acknowledged at the organizational level, a fundamental micro-level question remains understudied. How do employees form deep psychological connections with data? And how do such connections translate into employee innovative behavior (EIB)? Addressing these questions requires moving beyond dominant macro-level research perspectives. It is essential to explore the psychological transformation process when employees regard data as personal resources.

Existing studies have explored the above issues along two separate lines, yet fail to integrate them systematically. First, perceived data value (PDV) refers to employees’ subjective judgment on the functional, strategic and informational benefits brought by data. It acts as a key cognitive prerequisite for employees’ data-related behaviors (Förster et al., 2022). Second, digital leadership (DL) features digital vision communication, role modeling of data-driven behaviors and digital culture cultivation (Zeike et al., 2019). Empirical evidence shows that it effectively improves organizational innovation performance (Benitez et al., 2022) and employee creativity (Öngel et al., 2023). It also facilitates open innovation via intermediate pathways such as knowledge sharing (Fatima and Masood, 2024). Nevertheless, a paradox commonly exists in organizational practice. Even when employees recognize the great value of data and receive support from DL, they still show low engagement in data work, passive data usage and insufficient innovative application of data (Mikalef and Gupta, 2021). This gap between value perception and behavioral transformation indicates that PDV and external leadership support cannot directly stimulate EIB. Certain critical psychological mediating mechanisms remain underexplored. This theoretical gap serves as the core motivation for the present research.

Current literature presents systematic deficiencies across three interrelated dimensions. All these gaps point to an unresolved theoretical question. First, psychological ownership theory (POT) (Pierce et al., 2001, 2003) has been extended to various targets. These targets include jobs (Van Dyne and Pierce, 2004), organizations (O’Driscoll et al., 2006) and knowledge (Peng, 2013). Recent studies also prove that psychological ownership acts as a vital mediator in shaping innovative behaviors (Kousina and Voudouris, 2023). Even so, the construct of data asset psychological ownership (DAPO) for digital assets has rarely been discussed in existing research. Data differs fundamentally from traditional ownership objects. It features intangibility, replicability and ambiguous boundaries (Teece, 2018). A theoretically valuable question remains unanswered. Whether and how employees develop a sense of “these data belong to me” in digital work settings still needs exploration. Second, the micro psychological paths linking PDV to EIB remain unclear. Most existing studies on data value focus on organizational or strategic macro analysis (Verhoef et al., 2021). They largely ignore how individual employees translate PDV into concrete innovative actions. The cognitive-affective-behavior transition chain requires further clarification. Third, ample studies have confirmed the positive effect of DL on innovation (Benitez et al., 2022; Fatima and Masood, 2024; Öngel et al., 2023). However, few studies have systematically constructed relevant theories or conducted direct empirical tests on its moderating effect. Specifically, research is lacking on how DL regulates the transition from employees’ data perception to psychological ownership. It still remains unknown whether leadership context strengthens or weakens this psychological conversion process. Combined, the three research gaps raise a core research question. In digital work contexts, how does PDV translate into EIB via DAPO? What moderating role does DL play throughout this process? Data literacy refers to the ability to understand and use data to inform decisions (Mandinach and Gummer, 2013); data self-efficacy concerns individuals’ confidence in performing data-related tasks (Bandura, 1997); and data territoriality reflects exclusionary and defensive behaviors toward data (Brown et al., 2005). By contrast, the core of DAPO lies in employees’ positive sense that “this data is mine,” wherein the target of ownership becomes part of the extended self (Pierce et al., 2003).

To address the above questions systematically, this study integrates POT (Pierce et al., 2001) and conservation of resources (COR) theory (Hobfoll, 1989). It develops and tests a moderated mediation model. In this model, DAPO serves as the mediator, and DL acts as the boundary condition. When employees perceive high strategic value of data, they will actively devote cognitive resources to in-depth exploration. Gradually, they develop a sense of psychological ownership toward data through continuous personal engagement. POT theory further explains that DAPO motivates employees to safeguard data and pursue self-extension. It thus provides steady psychological impetus for EIB (Pierce et al., 2003). Through vision sharing and behavioral modeling, DL consolidates employees’ cognition of data value. It also improves the conversion efficiency from PDV to DAPO. Therefore, DL functions as a critical contextual factor in this psychological pathway.

This study makes theoretical contributions in three major aspects. First, this paper proposes and defines the new construct of DAPO. It extends the application scope of POT from traditional tangible resources and knowledge assets to data, the core element of the digital economy. This responds to scholars’ call to expand the contextual application of ownership theory (Pierce and Jussila, 2011). It also establishes a new psychological framework for interpreting employees’ engagement with digital resources. Second, this study constructs a sequential path: PDV—DAPO—EIB. It systematically uncovers the micro psychological mechanism linking data perception to innovative outcomes in digital settings. The findings strengthen the explanatory power of COR theory in digital work environments (Hobfoll, 1989). They also enrich research on the antecedents of EIB (Scott and Bruce, 1994). Third, this study verifies the positive moderating effect of DL on the association between PDV and DAPO. It identifies a new boundary condition for research on DL mechanisms. It also expands literature regarding leadership as an enabler of employees’ psychological resource accumulation (Benitez et al., 2022; Fatima and Masood, 2024). This research also yields practical implications for organizations undergoing digital transformation. Cultivating employees’ PDV and fostering their DAPO are two effective approaches to boost data-driven innovation. Enhancing leaders’ digital capabilities serves as a vital organizational investment to amplify the above effects.

## 2 Literature review and research hypotheses

### 2.1 Theoretical framework

This study takes POT and COR theory as two core theoretical foundations. It introduces DL as a key contextual variable and develops an integrated theoretical framework. POT was formally established by Pierce et al. (2001, 2003). Its core argument holds that individuals develop a subjective sense of “this is mine” toward a certain target. They integrate the target into their self-concept and regard it as part of the psychological self. This sense of belonging develops through three pathways. They are perceived control over the target, in-depth understanding, and continuous personal investment (Pierce and Jussila, 2011). Once individuals form psychological ownership of a target, they will generate protection motivation, self-efficacy and self-extension motivation. These factors further promote positive attitudes and behaviors (Avey et al., 2009; Kousina and Voudouris, 2023). The two theories complement each other well for the core arguments of this study. COR theory explains why and how employees actively invest resources to build connections with data after recognizing its high value. It elaborates the motivation mechanism behind the link from value perception to resource investment. POT theory further clarifies how such resource investment evolves into a sense of psychological belonging to data. It also illustrates how this belonging ultimately stimulates EIB. Together, they lay a solid theoretical foundation for the core pathway: PDV—DAPO—EIB. DL is embedded into the above pathway as a critical organizational contextual factor. It shapes employees’ cognition of data through vision communication and behavioral modeling (Benitez et al., 2022; Fatima and Masood, 2024). It also creates a supportive environment for employees to engage deeply with data via tool empowerment and organizational support (Öngel et al., 2023). DL strengthens the transformation from PDV to psychological belonging. It also exerts an overall moderating effect on the full mediating chain. For these reasons, it serves as the core boundary condition of the moderated mediation model in this research.

### 2.2 PDV and DAPO

PDV refers to employees’ subjective perception and evaluation of the functional utility, strategic benefits and informational value of data at work (Wixom and Ross, 2017). This perception is not a simple reflection of the objective attributes of data. Instead, employees actively interpret the potential benefits of data based on their work experience and cognitive frameworks. Thus, it varies greatly across individuals and contexts.

COR theory explains the relationship between PDV and DAPO. When employees perceive high strategic value of data, they classify data as a key resource worthy of active acquisition and protection (Hobfoll, 1989). This motivation drives employees to interact with data more frequently. They devote substantial cognitive resources to in-depth exploration and continuous use of data. In this process, employees gain rich data knowledge and a stronger sense of control over data. These conditions facilitate the three formation pathways of psychological ownership, namely perceived control, in-depth understanding and personal investment. POT theory further suggests that the formation of psychological ownership largely depends on individuals’ recognition and internalization of the target’s value (Pierce et al., 2001). If employees acknowledge the substantial value of data, they will treat data as a valuable target for personal investment. Through repeated contact, exploration and application, employees gradually incorporate data into their extended self-concept and develop a stable sense of psychological belonging. Recent studies also confirm that value judgment of resources acts as a core cognitive prerequisite for psychological ownership (Yin et al., 2020). These findings support the above theoretical reasoning.

*H1*: PDV is positively associated with DAPO. Specifically, higher PDV corresponds to stronger DAPO.

### 2.3 DAPO and EIB

EIB refers to a complete behavioral process in the workplace. It includes generating new ideas, seeking support for ideas, and putting ideas into practice. This process consists of three progressive stages: idea generation, idea promotion and idea implementation (Scott and Bruce, 1994; Janssen, 2000).

POT theory offers direct theoretical support for the link between DAPO and EIB. Pierce et al. (2003) stated that psychological ownership triggers three interrelated motivations. These include efficacy motivation to explore innovative uses of the target, identity motivation to demonstrate personal competence via the target, and belonging motivation to engage deeply and continuously with the target. When employees develop strong DAPO, these motivations work together. They tend to explore diverse applications of data and attempt disruptive data-driven innovation, thus showing more active innovative behavior. COR theory provides additional explanations. DAPO serves as a stable form of psychological capital (Hobfoll, 2001). It reduces employees’ fear of failure and resource loss during innovative exploration. It also maintains sufficient psychological security to sustain proactive innovative actions. Recent empirical studies further prove that psychological ownership positively predicts innovative behavior by boosting employees’ sense of responsibility and self-efficacy (Kousina and Voudouris, 2023; Yin et al., 2020). Conceptually, DAPO differs from related constructs. Unlike *data literacy* (Mandinach and Gummer, 2013) or *data self-efficacy* (Bandura, 1997), which denote capability and confidence, DAPO captures an affective sense of possession and identity attachment. Unlike *data stewardship* (Chen et al., 2015), which is role-prescribed, DAPO is self-oriented and psychologically internalized. Unlike *knowledge ownership* (Peng, 2013), which concerns codified knowledge, DAPO targets intangible digital assets (Teece, 2018). Unlike *data territoriality* (Brown et al., 2005), which entails defensive exclusion, DAPO emphasizes legitimate belonging and efficacy rather than restrictive control.

*H2*: DAPO is positively associated with EIB. That is, higher levels of DAPO lead to more active EIB.

### 2.4 The mediating role of DAPO

Drawing on the above arguments, this study proposes that DAPO mediates the relationship between PDV and EIB. PDV acts as a cognitive prerequisite for stimulating innovative behavior. However, pure value judgment at the cognitive level cannot directly translate into practical innovative actions. Employees need to complete an affective transition from value perception to psychological belonging. They gradually integrate data into their extended self-concept. This process fosters stable motivation to explore data actively and take innovative risks (Pierce et al., 2001).

Drawing on the resource caravan perspective within COR theory (Hobfoll et al., 2018), PDV motivates employees to invest cognitive resources. Continuous resource accumulation strengthens their DAPO. As a form of psychological capital, DAPO provides solid psychological security for employees to engage in innovation. This resource transition path, namely cognitive resource investment—psychological resource possession—behavioral resource release, clarifies the internal mechanism of how PDV influences innovative behavior. Recent research also indicates that perceived value of job resources can predict innovative performance only through the mediation of individual psychological states (Zhang et al., 2018). This conclusion applies equally to the data-oriented context of the present study.

*H3*: DAPO mediates the relationship between PDV and EIB. Specifically, PDV is associated with EIB through DAPO.

### 2.5 The moderating role of DL

PDV facilitates the formation of DAPO. Yet this transition from cognition to psychological belonging does not work equally well under all leadership conditions. As a key organizational factor, leadership context exerts notable moderating effects on employees’ psychological states (Liu et al., 2012; Zhang and Bartol, 2010). This study argues that DL positively moderates the link between PDV and DAPO. Two complementary theoretical perspectives explain its underlying mechanisms.

From the perspective of social learning theory (Bandura, 1986), leaders demonstrate data-driven decision-making and advocate the value of data. They set clear and credible behavioral models for employees. Under strong DL, employees gain self-efficacy in data use from leaders’ behaviors. They also deepen their recognition of data value through consistent advocacy. In turn, they are more willing to invest efforts in data-related work, which accelerates the development of DAPO (Benitez et al., 2022; Öngel et al., 2023). From the perspective of social exchange theory (Blau, 1964), DL provides digital tools, data empowerment and a fault-tolerant climate for innovation. It builds a supportive environment for employees to engage deeply with data. Employees who receive such support develop a reciprocal mindset. They recognize the legitimate value of data resources and devote more efforts to data work. This process further strengthens their DAPO (Fatima and Masood, 2024). By contrast, weak DL means insufficient organizational support and behavioral guidance. Even if employees acknowledge data value, they tend to reduce relevant investment. The conversion from PDV to DAPO will be weakened accordingly.

*H4*: DL positively moderates the association between PDV and DAPO. The positive association becomes stronger when the level of DL rises.

### 2.6 The moderated mediation effect of DL

This study integrates mediating and moderating pathways to construct a moderated mediation model. DL not only moderates the effect of PDV on DAPO, but also generates an overall facilitating effect on the full mediating chain: PDV-DAPO-EIB.

Under high levels of DL, employees receive clear digital vision and sufficient organizational resources. They can convert PDV into strong psychological belonging to data more effectively. The enhanced DAPO further stimulates employees’ innovation motivation. As a result, the indirect effect of PDV on EIB via DAPO is significantly amplified. On the contrary, low DL hinders the transition from PDV to DAPO. The mediating effect of DAPO is weakened, and the indirect influence of PDV on innovative behavior is diminished accordingly. In line with the resource gain spiral logic of COR theory (Hobfoll et al., 2018), contextual resources provided by DL include digital vision, tool support and empowerment climate. These resources act as external catalysts for employees’ engagement with data. They streamline the resource accumulation chain driven by PDV, namely cognitive resource investment, psychological belonging and innovative outcomes. A stronger positive resource gain spiral is therefore formed. Prior studies have confirmed that leadership behaviors moderate the indirect paths from resource perception to innovative behavior via psychological mechanisms (Fatima and Masood, 2024; Zhang et al., 2018). These findings support the theoretical reasoning of the moderated mediation model.

**Figure 1:**
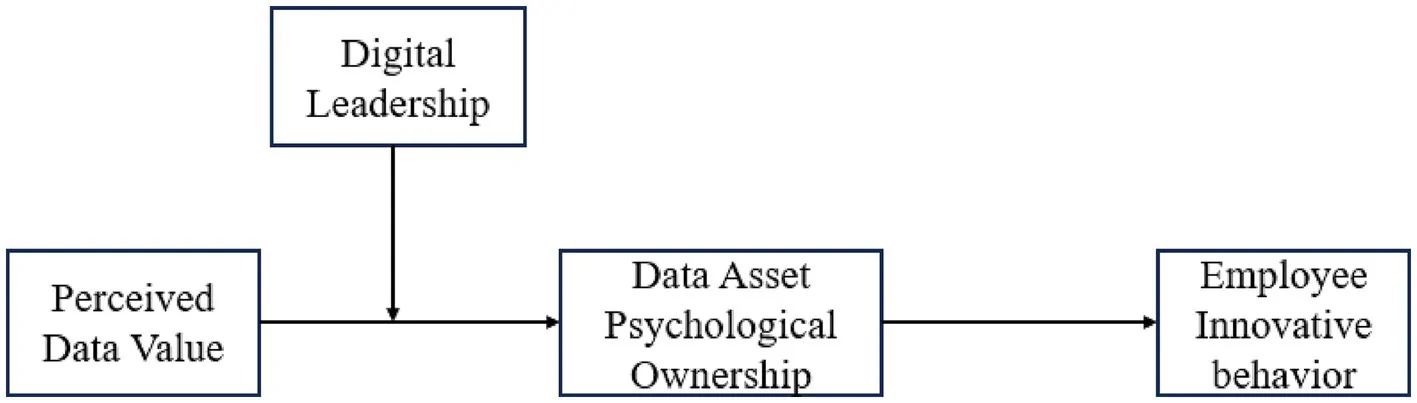
Theoretical model.

*H5*: DL positively moderates the indirect effect of PDV on EIB via DAPO. The indirect effect of PDV on EIB through DAPO becomes stronger at higher levels of DL. This pattern verifies the moderated mediation model. (Figure 1)

## 3 Methods

### 3.1 Sample and data collection

This study adopted a three-wave time-lagged research design. The Credamo online platform, known for its stringent data quality control mechanisms and extensive application in social science and behavioral research, was utilized for data collection. This platform is widely adopted for both corporate and academic surveys in China. The whole data collection lasted 5 weeks, from March 2 to April 5, 2026. There was a 2-week interval between each wave. This time-lag design helped reduce common method bias and capture dynamic relationships among variables.

At Time 1 (March 2, 2026), the first questionnaire was distributed. It measured participants’ demographic information, including gender, education, age, work tenure, job rank and job type. It also assessed the core independent variable, PDV and Moderating variable, DL. Questionnaires were distributed to enterprise employees through the sample pool service of Credamo. A total of 400 responses were collected. After preliminary screening, 370 valid responses were retained to form the Time 1 sample pool. Two weeks later, the second-wave survey was delivered to the Time 1 participants at Time 2 (March 16, 2026). This survey mainly measured the mediator variable, DAPO. Moderate incentives were provided to maintain participant engagement. We obtained 335 valid responses in this phase. After another 2 weeks, the third-wave questionnaire was released to the Time 2 participants at Time 3 (March 30, 2026). It measured the dependent variable (EIB). All data collection was completed on April 5, 2026, with 305 valid responses finally retrieved.

All three waves of data were matched and integrated using a unique built-in identification code for each participant. We further eliminated invalid questionnaires according to four criteria. First, responses completed in less than 120 s in any single wave. Second, responses that failed embedded attention check items. Third, answers with obvious logical inconsistencies on reverse-coded items. Fourth, responses with uniform or regular alternating answer patterns. After strict screening, 279 valid matched samples were obtained. The overall valid response rate reached 68.75%. This study used convenience sampling. Participants were full-time employees from enterprises across different regions and industries in China. This approach improved the diversity and representativeness of the sample.

### 3.2 Measures

A 5-point Likert scale was employed for all variable measurements, with responses anchored from 1 (strongly disagree) to 5 (strongly agree). To ensure conceptual consistency between the English and Chinese versions of the scales, the standard translation and back-translation procedure recommended by Brislin (1980) was followed.

*PDV*: Guided by the theoretical framework, this study initially developed a scale for PDV. The original scale contained nine items derived from three theoretical perspectives: perceived importance, perceived scarcity and perceived irreplaceability. The perceived importance dimension was adapted from the perceived usefulness scale developed by Deng et al. (2004). Rooted in the Technology Acceptance Model, this scale assesses how individuals judge resources based on work performance improvement. We applied its logic to the context of data assets. The perceived scarcity dimension was developed based on Resource Dependence Theory (Pfeffer and Salancik, 1979). This theory states that less accessible resources bring greater influence to their holders. We used this perspective to evaluate employees’ subjective judgments on the accessibility of data assets within organizations. The perceived irreplaceability dimension drew on the knowledge uniqueness scale of Hinds et al. (2001). This scale confirms that irreplaceable and hard-to-replicate knowledge shapes employees’ organizational status. We extended this measurement logic to data assets.

A panel of experts reviewed all items. Two representative items were selected from each of the three theoretical perspectives, leaving six items in total. Classical Test Theory (CTT) was adopted for item reduction and factor structure examination to verify construct validity. To avoid potential overestimation of model fit arising from conducting exploratory factor analysis (EFA) and confirmatory factor analysis (CFA) on the same sample, data were collected in two separate waves. The first wave yielded 200 valid responses for EFA, while the second independently collected sample comprised 279 valid responses for CFA. We first conducted EFA on the first-wave sample (*n* = 200) with principal component analysis and varimax rotation. The results showed a KMO value of 0.838 and a significant Bartlett’s test of sphericity (*χ*^2^ = 575.49, df = 15, *p* < 0.001). These values indicated the dataset was suitable for factor analysis. Only one factor had an eigenvalue above 1, which explained 59.96% of the total variance. Factor loadings of all items ranged from 0.644 to 0.843. One item with a low factor loading (below 0.50) and ambiguous theoretical connotation was removed according to standard item selection criteria. The remaining five items formed a stable unidimensional structure. Although the initial items were derived from three distinct theoretical perspectives (importance, scarcity, and irreplaceability), the EFA results indicated that these perspectives empirically converged into a single factor at the employee level of analysis, suggesting that employees tend to integrate these facets into a holistic value judgment rather than differentiating them cognitively (Zeithaml, 1988). Accordingly, we retained the label perceived data value (PDV) for this unidimensional construct. CFA was further performed on the second independent sample (*n* = 279) to validate the stability of this unidimensional structure. The model achieved satisfactory fit indices: *χ*^2^/df = 2.243, GFI = 0.985, AGFI = 0.955, CFI = 0.990, TLI = 0.979, RMSEA = 0.067. All indices met widely accepted criteria (CFI > 0.90, RMSEA < 0.08), supporting acceptable construct validity of the unidimensional structure. The standardized factor loadings of the five items ranged from 0.539 to 0.834, all exceeding the recommended threshold of 0.50 and statistically significant (*p* < 0.001). Composite reliability (CR) was 0.857, and average variance extracted (AVE) was 0.550, exceeding the recommended thresholds of 0.70 and 0.50, respectively (Hair et al., 2019), indicating adequate convergent validity. The final five items were rated on a 5-point Likert scale (1 = strongly disagree, 5 = strongly agree): 1. The data assets I master are critical to my daily work performance. 2. The data assets I master cannot be easily replaced by other resources. 3. The value provided by my data is unique and difficult to replicate. 4. Few colleagues have access to the data resources I hold. 5. The data assets I master are scarce within the organization. Reliability analysis yielded a Cronbach’s *α* of 0.852, indicating good internal consistency of the scale.

*DL:* This construct was measured with a revised scale from Zeike et al. (2019). The original scale captures employees’ perceptions of their direct supervisors regarding digital awareness, technical proficiency and digital transformation promotion. The revised version includes six items covering digital enthusiasm, digital skills, continuous learning of digital knowledge, transformation advocacy, motivation mobilization, and understanding of organizational structures and processes. The Cronbach’s *α* coefficient of this scale was 0.864 in the current study.

*DAPO*: This variable was measured using a revised scale developed by Van Dyne and Pierce (2004). The original seven-item scale assesses psychological ownership toward organizations. It consists of three dimensions: sense of belonging, self-identity and perceived efficacy, with a reliability coefficient of 0.91. Following the revision approach adopted by Peng (2013) in the research on knowledge psychological ownership, we replaced the original referent “organization” with “data assets I use in my work.” This adjustment adapted the scale to the research context of data assets. The revised scale retains all seven items across the three original dimensions. The sense of belonging dimension includes three items, such as “I feel the data assets I use at work are mine.” The self-identity dimension contains two items, such as “These data assets reflect who I am.” The perceived efficacy dimension has two items, such as “I feel I have full control over these data assets.” The adapted scale captures legitimate psychological ownership rather than data territoriality or restrictive control (Peng, 2013). The item “I feel I have full control” reflects a sense of efficacy and mastery, not exclusionary gatekeeping. In the present study, the Cronbach’s *α* coefficient of this scale was 0.933.

*EIB*: This study adopted the EIB scale developed by Scott and Bruce (1994). The original six-item scale evaluates individual innovative behavior in the workplace. It covers three dimensions: idea generation, idea promotion and idea implementation. The specific items are as follows: I look for ideas related to new technologies, workflows and products. I often come up with creative ideas. I present new ideas to my team and organization to gain recognition. I acquire necessary resources to put new ideas into practice. I develop sound plans to launch new products or services. Overall, I consider myself an innovative person. The Cronbach’s α coefficient of this scale was 0.818 in this study.

*Control Variables*: Following prior research (Bellmann and Hübler, 2022; Cadario et al., 2023), we controlled for demographic and work-related variables that may affect employees’ data-related psychology and behaviors. These variables were coded as follows: Gender; Educational level; Age; Work tenure; Job rank; Job type.

### 3.3 Analytical strategy

First, we conducted CFA via AMOS 29.0. We compared competing measurement models to examine the discriminant validity of four core variables, namely PDV, DAPO, DL and EIB. Model fit was also evaluated in this step. Second, SPSS was used for descriptive statistics. We calculated the mean, standard deviation, Cronbach’s α and Pearson correlation coefficients for all variables. These results provided preliminary evidence for subsequent hypothesis testing. Third, we adopted the PROCESS macro for SPSS (Version 3.3, Model 4 and Model 7) to test the research hypotheses. We examined the mediating effect of DAPO using Model 4. We further tested the moderating effect of DL and the first-stage moderated mediation effect. Prior to constructing the interaction term (PDV*DL), all continuous variables (PDV and DL) were mean-centered to reduce nonessential multicollinearity. The bias-corrected Bootstrap method with 5,000 resamples was applied to generate confidence intervals. These intervals determined the statistical significance of all hypothesized effects.

## 4 Results

### 4.1 Confirmatory factor analysis

We performed CFA using AMOS 29.0 to assess the discriminant validity of four core variables: PDV, DAPO, DL and EIB. We established and compared several competing models, including the four-factor model, three-factor models, two-factor model and single-factor model. Model fit indices are presented in Table 1. The four-factor model yielded the best fit. Its indices were *χ*^2^/df = 1.992 (<3), CFI = 0.936, TLI = 0.928 (>0.90), RMSEA = 0.060 (<0.08), and SRMR = 0.059 (<0.08). All values met or exceeded the recommended criteria (Hu and Bentler, 1999). This model also outperformed all alternative models, whose CFI values were below 0.90 and RMSEA values exceeded 0.08. These findings support satisfactory discriminant validity among the four variables. Standardized factor loadings of all items ranged from 0.502 to 0.892, and all were statistically significant (*p* < 0.001). Specifically, factor loadings for PDV ranged from 0.539 to 0.834. Loadings for DL fell between 0.687 and 0.779. Loadings for DAPO were from 0.700 to 0.892. Loadings for EIB ranged from 0.502 to 0.727. All loadings exceeded the threshold of 0.50 (Hair et al., 2019). The results indicate adequate convergent validity of all scales.

**Table 1:** Comparison of fit indices across confirmatory factor analysis models.

Model	*χ* ^2^	df	*χ*^2^/df	CFI	TLI	RMSEA	SRMR
Four-factor model (PDV, DAPO, DL, EIB)	489.992	246	1.992	0.936	0.928	0.060	0.059
Three-factor model (PDV + DAPO, DL, EIB)	900.510	249	3.617	0.828	0.810	0.097	0.091
Two-factor model (PDV + DAPO+DL, EIB)	1465.660	251	5.839	0.680	0.648	0.132	0.123
Single-factor model (PDV + DAPO+DL + EIB)	1665.853	252	6.611	0.627	0.592	0.142	0.123

*N*, 279; PDV, Perceived Data Value; DAPO, Data Asset Psychological Ownership; DL, Digital Leadership; EIB, Employee Innovative Behavior.

### 4.2 Common method bias

We first adopted Harman’s single-factor test to assess common method bias. An EFA was conducted on all key variables without rotation. The first factor with an eigenvalue greater than 1 explained 39.422% of the total variance. This value was below the critical threshold of 40% (Podsakoff et al., 2003), indicating that common method bias did not substantially affect the results. We further conducted the unmeasured latent method factor test. We first established a baseline four-factor model (M1). Then we added one latent common method factor to construct Model 2 (M2). We compared the fit indices between M1 and M2. The differences were ∆*χ*^2^/df = 0.146, ∆CFI = 0.001, ∆TLI = 0.001, ∆RMSEA = 0.005. The changes in CFI and TLI were both less than 0.01, and the change in RMSEA was below 0.05. These results suggest that adding the common method factor did not substantially improve model fit. Taken together, although common method bias cannot be completely ruled out, these tests suggest that it is unlikely to pose a serious threat to the validity of our finding.

### 4.3 Correlation analysis

Table 2 presents the correlation results. PDV was positively correlated with DL (*r* = 0.435, *p* < 0.01), DAPO (*r* = 0.521, *p* < 0.01) and EIB (*r* = 0.519, *p* < 0.01). DL showed significant positive correlations with DAPO (*r* = 0.441, *p* < 0.01) and EIB (*r* = 0.695, *p* < 0.01). DAPO was also positively associated with EIB (*r* = 0.494, *p* < 0.01). These correlation findings provide preliminary evidence for the subsequent hypothesis testing.

**Table 2:** Means, standard deviations and correlation coefficients of variables (*N* = 279).

Variables	*M*	SD	1	2	3	4	5	6	7	8	9
1. Gender	1.560	0.498	1								
2. Edu	3.050	0.598	0.022	1							
3. Age	1.900	0.863	−0.037	−0.054	1						
4. Exp	3.240	1.069	−0.039	−0.052	0.810^**^	1					
5. Pos	1.910	0.952	0.025	0.232^**^	0.379^**^	0.438^**^	1				
6. Job	1.480	0.501	0.188^**^	−0.089	0.096	0.135^*^	−0.056	1			
7. PDV	3.842	0.661	−0.121^*^	0.199^**^	−0.018	−0.032	0.164^**^	−0.392^**^	1		
8. DL	3.947	0.667	−0.089	0.054	0.088	0.043	0.185^**^	−0.380^**^	0.435^**^	1	
9. DAPO	3.467	0.937	−0.109	0.126^*^	0.003	−0.032	0.134^*^	−0.269^**^	0.521^**^	0.441^**^	1
10. EIB	4.090	0.525	−0.072	0.186^**^	0.049	0.050	0.295^**^	−0.289^**^	0.519^**^	0.695^**^	0.494^**^

^*^p < 0.05, ^**^p < 0.01.

### 4.4 Hypothesis testing

Hierarchical regression analysis was used to test the research hypotheses. The regression results are presented in Table 3. To eliminate multicollinearity bias, VI*F* values of key variables were computed. The maximum VIF was 3.188, well below the cutoff value of 5, suggesting no severe multicollinearity issue. As shown in Model 1 of Table 3, PDV significantly and positively predicted DAPO (*β* = 0.675, *p* < 0.001). After controlling for demographic variables, PDV explained a significant proportion of variance in DAPO. Hypothesis 1 was supported. Model 4 indicated that PDV had a significant positive effect on EIB (*β* = 0.346, *p* < 0.001). In Model 6, after adding the mediator DAPO, the direct effect of PDV on EIB remained significant (*β* = 0.237, *p* < 0.001). Meanwhile, DAPO significantly predicted EIB (*β* = 0.161, *p* < 0.001). Hypothesis 2 was supported. The Bootstrap method was adopted to further examine the mediating effect of DAPO for Hypothesis 3. As shown in Table 4, the total effect of PDV on EIB was 0.393, with a 95% confidence interval of [0.291, 0.495] excluding zero. The direct effect was 0.285, with a 95% confidence interval of [0.195, 0.375]. The indirect effect via DAPO was 0.108, with a 95% confidence interval of [0.058, 0.164] excluding zero. The relative mediating effect size was 27.5%. These results revealed that DAPO exerted a partial mediating effect. Hypothesis 3 was supported.

**Table 3:** Results of regression analysis.

Variables	DAPO												EIB											
	Model 1				Model 2				Model 3				Model 4				Model 5			Model 6				
	*β*	SE	*t*	*p*	*β*	SE	*t*	*p*	*β*	SE	*t*	*p*	*β*	SE	*t*	*p*	*β*	SE	*t*	*p*	*β*	SE	*t*	*p*
Gender	−0.136	0.111	−1.225	0.222	−0.080	0.099	−0.809	0.419	−0.095	0.095	−1.004	0.316	−0.037	0.060	−0.616	0.538	−0.008	0.054	−0.154	0.878	0.005	0.052	0.089	0.930
Edu	0.120	0.096	1.252	0.212	0.016	0.086	0.186	0.853	0.026	0.083	0.319	0.750	0.089	0.051	1.723	0.086	0.035	0.047	0.754	0.451	0.033	0.045	0.731	0.466
Age	0.074	0.107	0.691	0.490	0.072	0.095	0.758	0.449	0.042	0.091	0.465	0.643	−0.001	0.057	−0.011	0.991	−0.001	0.052	−0.026	0.979	−0.013	0.050	−0.261	0.795
Exp	−0.097	0.090	−1.082	0.280	−0.081	0.080	−1.011	0.313	−0.070	0.077	−0.909	0.364	−0.015	0.048	−0.307	0.759	−0.007	0.044	−0.152	0.879	0.006	0.042	0.153	0.879
Pos	0.126	0.066	1.901	0.058	0.065	0.060	1.091	0.276	0.041	0.057	0.717	0.474	0.150^***^	0.036	4.218	<0.001	0.119^***^	0.032	3.667	<0.001	0.109^***^	0.031	3.486	<0.001
Job	−0.437^***^	0.112	−3.903	<0.001	−0.120	0.107	−1.118	0.265	0.087	0.110	0.791	0.430	−0.266^***^	0.060	−4.424	<0.001	−0.104	0.058	−1.781	0.076	−0.085	0.056	−1.512	0.132
PDV					0.675^***^	0.081	8.304	<0.001	0.631^***^	0.087	7.276	<0.001					0.346^***^	0.044	7.822	<0.001	0.237^***^	0.047	5.009	<0.001
DAPO																					0.161^***^	0.032	5.078	<0.001
DL									0.427^***^	0.083	5.115	<0.001												
PDV*DL									0.234^*^	0.084	2.766	<0.05												
*R* ^2^	0.101				0.284				0.352				0.173				0.325			0.384				
*F*	5.104^**^				15.319^**^				16.215^**^				9.491^**^				18.675^**^			21.059^**^				

^*^p < 0.05, ^**^p < 0.01; ^***^p < 0.001.

**Table 4:** Total effect, direct effect and mediating effect.

Effect type	Effect size	Standard error	95% CI lower	95% CI upper	Relative effect size
Total effect	0.393	0.052	0.291	0.495	
Direct effect	0.285	0.046	0.195	0.375	72.5%
Indirect effect	0.108	0.027	0.058	0.164	27.5%

**Figure 2:**
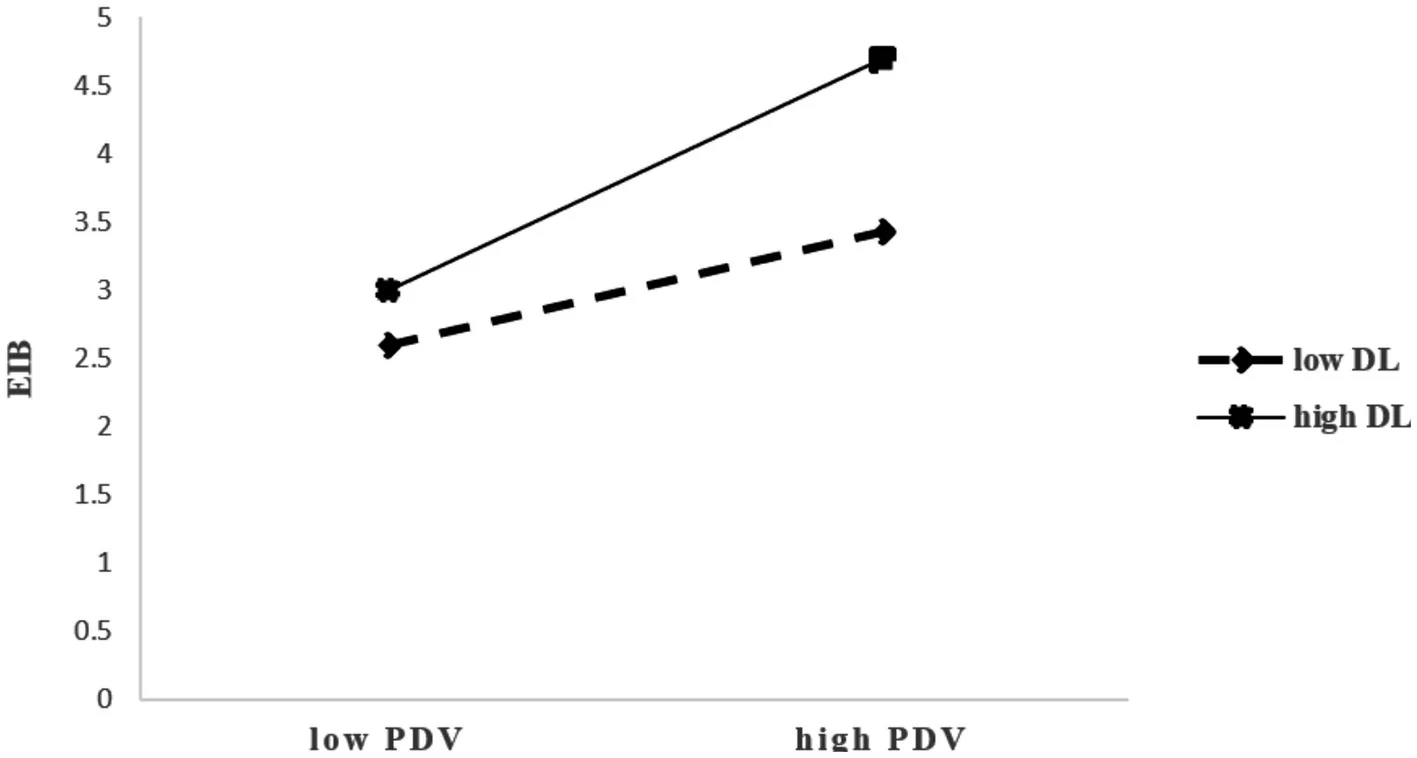
Moderating effect plot of DL.

To test the moderating effect of DL, we added the main effect of DL and the interaction term between PDV and DL based on Model 2 to form Model 3. Model 3 showed that the interaction term significantly predicted DAPO (*β* = 0.234, *p* < 0.05). This finding confirmed the moderating role of DL, so Hypothesis 4 was supported. This model yielded *R*^2^ = 0.352 with a significant F value (*p* < 0.01), and it explained more variance than Model 2 (*R*^2^ = 0.284). A simple slope plot was drawn to clarify the direction of the moderating effect (see Figure 2). When DL was at a low level, the positive predictive effect of PDV on DAPO was weak. When DL was at a high level, this positive relationship became stronger. Simple slope tests further verified that the slope was significantly larger in the high DL group. Higher DL strengthened the positive influence of PDV on DAPO.

We then applied the Bootstrap method to calculate the indirect effect across different levels of DL. The index of moderated mediation was 0.037, with a Bootstrap standard error of 0.019 and a 95% Bootstrap confidence interval of [0.005, 0.074] excluding zero. The moderating effect on the mediating path was statistically significant. Specifically, at the low level of DL (one standard deviation below the mean), the indirect effect was 0.084 (BootSE = 0.025, 95% CI = [0.035, 0.138]). At the moderate level, the indirect effect was 0.108 (BootSE = 0.028, 95% CI = [0.059, 0.167]). At the high level of DL (one standard deviation above the mean), the indirect effect was 0.133 (BootSE = 0.033, 95% CI = [0.073, 0.206]). Pairwise comparison showed a significant difference in indirect effects between the two groups (difference = 0.050, 95% CI = [0.005, 0.101]). Overall, DL positively moderated the mediating effect of DAPO. The indirect influence of PDV on EIB through DAPO became stronger as DL increased. Hypothesis 5 was supported.

## 5 Discussion

Based on POT and COR theory, this study took DL as a key contextual variable and established a moderated mediation model. It systematically examined the relationships and underlying mechanisms among PDV, DAPO, EIB and DL. All five hypotheses were empirically supported. The findings reveal the psychological drivers and boundary conditions of EIB in digital work settings, and offer valuable theoretical insights and practical implications for human resource management and leadership practices in the digital economy. The results indicate that PDV is positively associated with DAPO, which is consistent with the core arguments of COR theory (Hobfoll, 1989). When employees regard data as a high-value work resource, they actively devote cognitive and time resources to build deep connections with data. Gradually, they develop a stable sense of “this is mine” toward data through accumulated control, in-depth understanding and continuous personal engagement (Pierce et al., 2001). This finding is consistent with the theoretical view that resource value perception serves as a core prerequisite for psychological ownership (Yin et al., 2020). It also extends the application scope of POT theory to data, a new type of digital production factor. Existing studies on psychological ownership mainly focus on traditional targets such as physical assets, jobs and organizations (Van Dyne and Pierce, 2004; Avey et al., 2009). This study includes data as a new target of psychological ownership. It suggests that employees can develop stable ownership feelings toward digital, intangible and dynamically evolving data resources, which broadens the applicable boundary of POT. In addition, DAPO exerts a significant positive effect on EIB. This result aligns with prior evidence regarding the positive link between psychological ownership and innovation (Kousina and Voudouris, 2023). In digital contexts, employees do not rely merely on external incentives to engage in innovation. A strong sense of psychological ownership over data can foster self-directed innovative activities. This logic echoes self-determination theory, which states that the need for autonomy fuels intrinsic motivation (Deci and Ryan, 2000).

In terms of mediating effects, this study finds that PDV cannot directly trigger innovative behavior. Instead, it needs to be translated via DAPO to facilitate innovative outcomes. This result is consistent with the resource transition chain: cognitive resource investment—psychological resource possession—behavioral resource release (Hobfoll et al., 2018). Perception of data value acts as the cognitive starting point for innovation. However, if such value judgment cannot be internalized into stable psychological ownership, an obvious implementation gap will emerge between value cognition and practical innovative actions. This finding is consistent with the conclusion of Zhang et al. (2018) that perceived value of job resources predicts innovative behavior only through psychological states. It also offers empirical insights into the internal psychological mechanism of realizing data value in digital contexts and fills the research gap in this field.

Regarding moderating effects and moderated mediation effects, this study reveals that DL positively moderates the transition from PDV to DAPO. The positive association between PDV and DAPO becomes stronger under high DL. By contrast, this transition is markedly weakened when DL is at a low level. This result integrates the theoretical perspectives of social learning theory and social exchange theory. From the perspective of social learning theory, leaders with strong DL demonstrate data-driven behaviors. They set clear and credible behavioral models for employees and accelerate the translation of data value perception into active engagement with data (Bandura, 1986; Öngel et al., 2023). From the perspective of social exchange theory, leaders provide digital tool support, data empowerment and a fault-tolerant climate for innovation. These supports trigger employees’ reciprocal motivation and strengthen their recognition of the legitimate value of data resources. Accordingly, PDV is more likely to translate into deep personal investment in data (Blau, 1964; Fatima and Masood, 2024). This finding is consistent with the view that DL facilitates innovative performance (Benitez et al., 2022). It further clarifies the underlying psychological mechanisms through which DL functions. The indirect path from PDV to EIB via DAPO is significantly enhanced under high DL, while it is diminished or even blocked under low DL. DL acts not only as a promoter but also as an amplifier in this mediating mechanism. It improves the efficiency of the resource gain spiral: resource value recognition—psychological resource accumulation—behavioral resource activation (Hobfoll et al., 2018). By reshaping how employees accumulate psychological resources, DL optimizes the full pathway from value perception to innovative outcomes.

### 5.1 Theoretical contributions

First, this study expands the object boundary of POT. Existing research on psychological ownership has long centered on traditional tangible objects such as jobs, organizations and knowledge. Few studies have systematically examined whether intangible and dynamic data resources in digital settings can serve as targets of psychological ownership. This study introduces data as a new research object into the empirical framework for the first time. It verifies that employees can develop stable ownership feelings toward data, and identifies a new research direction for POT in the digital economy era.

Second, this study integrates COR theory and POT to establish a comprehensive framework for explaining the psychological mechanisms of innovative behavior in digital contexts. Prior studies mostly focused on organizational data analytics capabilities or technical tools when exploring the link between data and employee behaviors, while paying limited attention to individual psychological processes. Taking the resource transition chain of cognitive resource investment—psychological resource possession—behavioral resource release as the core logic, this study combines the resource accumulation tenet of COR theory with the psychological belonging mechanism of POT theory via the key mediator of DAPO. It clarifies the underlying psychological pathway through which PDV affects EIB, and provides a new theoretical framework for research on innovation in digital work environments.

Third, this study enriches research on the micro psychological mechanisms of DL. Current literature on DL mainly explores its impacts on organizational outcomes, such as digital transformation performance and organizational innovation. Research on how DL functions at the individual psychological level remains insufficient. This study treats DL as the moderator in a moderated mediation model. It confirms that DL amplifies the whole mediating path of PDV—DAPO—EIB. The findings offer a novel micro psychological perspective for DL theory, and reveal its critical role as a situational catalyst in fostering employees’ psychological resource accumulation and innovation motivation.

### 5.2 Practical implications

First, organizations should focus on cultivating and improving employees’ PDV. The results indicate that PDV acts as the cognitive origin of DAPO and subsequent innovative behavior. Managers can systematically enhance employees’ awareness of the strategic value of data through data literacy training, sharing cases of data-driven decision-making, and visualizing outcomes of data application. These measures motivate employees to engage with data and lay a solid psychological foundation for data-driven innovation.

Second, build a working environment conducive to the development of DAPO. This construct serves as a vital psychological mediator linking PDV to innovative behavior. Companies may formulate institutional arrangements including data usage authorization, clear definition of data ownership, and sufficient autonomy for data exploration. Such policies enable employees to access, manage and utilize data fully. Continuous interaction with data helps foster a stable sense of psychological ownership and stimulates innovative potential.

Third, strengthen the development of managers’ DL. DL functions as a critical situational lever to amplify the overall effect of the pathway from PDV to DAPO and then to innovative behavior. Enterprises should incorporate DL into the core competency assessment and training system for managers. Priority should be given to improving their capabilities in communicating digital vision, demonstrating data-driven practices, and delivering organizational support for digital initiatives. A workplace with strong DL can fully activate the whole transformation chain from data value perception and psychological ownership to innovative outcomes, so as to maximize the value of digital talents and overall organizational innovation performance.

### 5.3 Limitations and future research

First, although this study employed a time-lagged design to temporally separate the measurement of the independent, mediating, and dependent variables, it is still fundamentally a correlational survey-based design. Therefore, it cannot definitively establish strict causal relationships among the variables as effectively as experimental designs or rigorous longitudinal studies (e.g., with repeated measures of the same variables). Future research could employ experimental designs or collect multi-wave, multi-source data to more rigorously test the dynamic causal links. Second, the samples were mainly collected from industries with advanced digital transformation. Thus, the generalizability of the findings across different contexts needs further verification. Follow-up research can retest the research model in enterprises with varying levels of digital maturity, industry backgrounds and organizational sizes, so as to systematically evaluate the external validity of the results. Third, although procedural controls such as time-lagged measurement and anonymous questionnaires were applied during data collection, all data were self-reported by employees. Common method bias may still exist to a certain degree (Podsakoff et al., 2003). Future research can mitigate this issue by incorporating supervisor ratings or objective performance indicators. Fourth, this study only examined a single pathway where PDV influences innovative behavior via DAPO. Subsequent studies can explore potential dark sides of DAPO, such as data territoriality and knowledge hiding. Other possible psychological mediators, including data self-efficacy and digital work engagement, can also be investigated. These efforts will help build a more comprehensive theoretical framework for innovative behavior in digital settings.

## 6 Conclusion

Based on POT and COR, this study constructed and verified a moderated mediation model, with DAPO as the mediator and DL as the moderator. The results suggest that PDV is associated with EIB through the mediating pathway of DAPO. DL appears to strengthen the mediating path, which aligns with the proposed theoretical framework. These findings not only extend the digital application scope of POT and integrate the explanatory frameworks of COR and POT theoretically, but also provide practical management guidelines for enterprises. Organizations can motivate EIB by improving employees’ recognition of data value, fostering their sense of data-related psychological ownership, and developing DL capabilities.

## Data Availability

The raw data supporting the conclusions of this article will be made available by the authors, without undue reservation.
